# Supplementary figures and images for: IL-1β knockdown inhibits cigarette smoke extract-induced inflammation and apoptosis in vascular smooth muscle cells
Source: PLoS One. 2023 Feb 15;18(2):e0277719. doi: 10.1371/journal.pone.0277719 (PMC9931126; doi:10.1371/journal.pone.0277719)

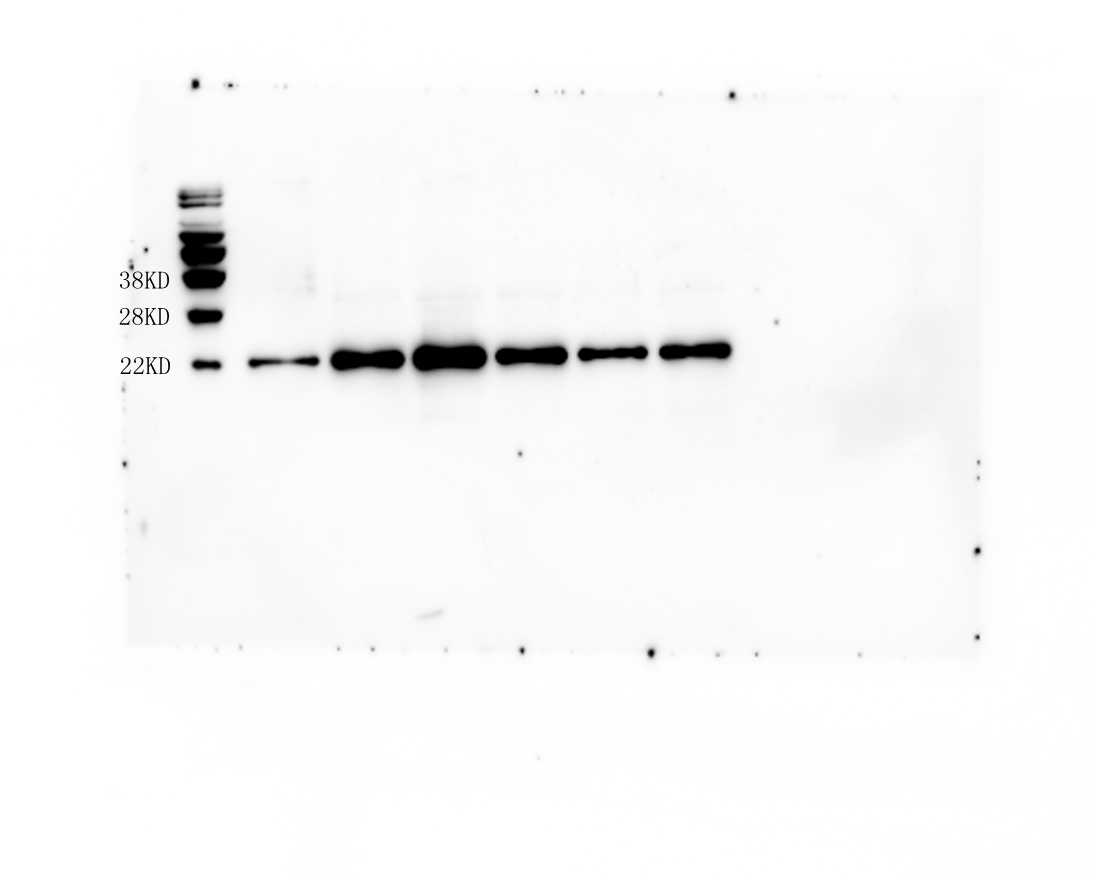

Supplement: S1 File — (ZIP) [file pone.0277719.s001.zip › original blot images/bax.jpg]

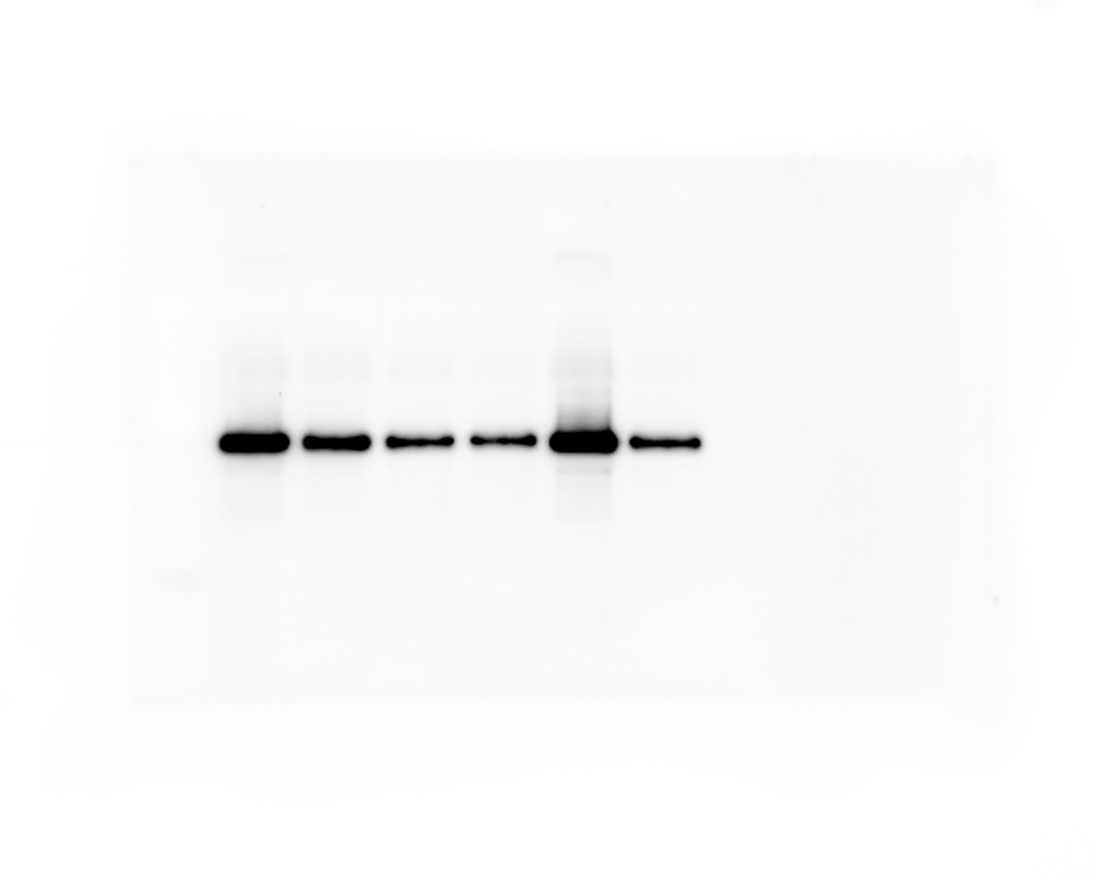

Supplement: S1 File — (ZIP) [file pone.0277719.s001.zip › original blot images/Bcl-2.jpg]

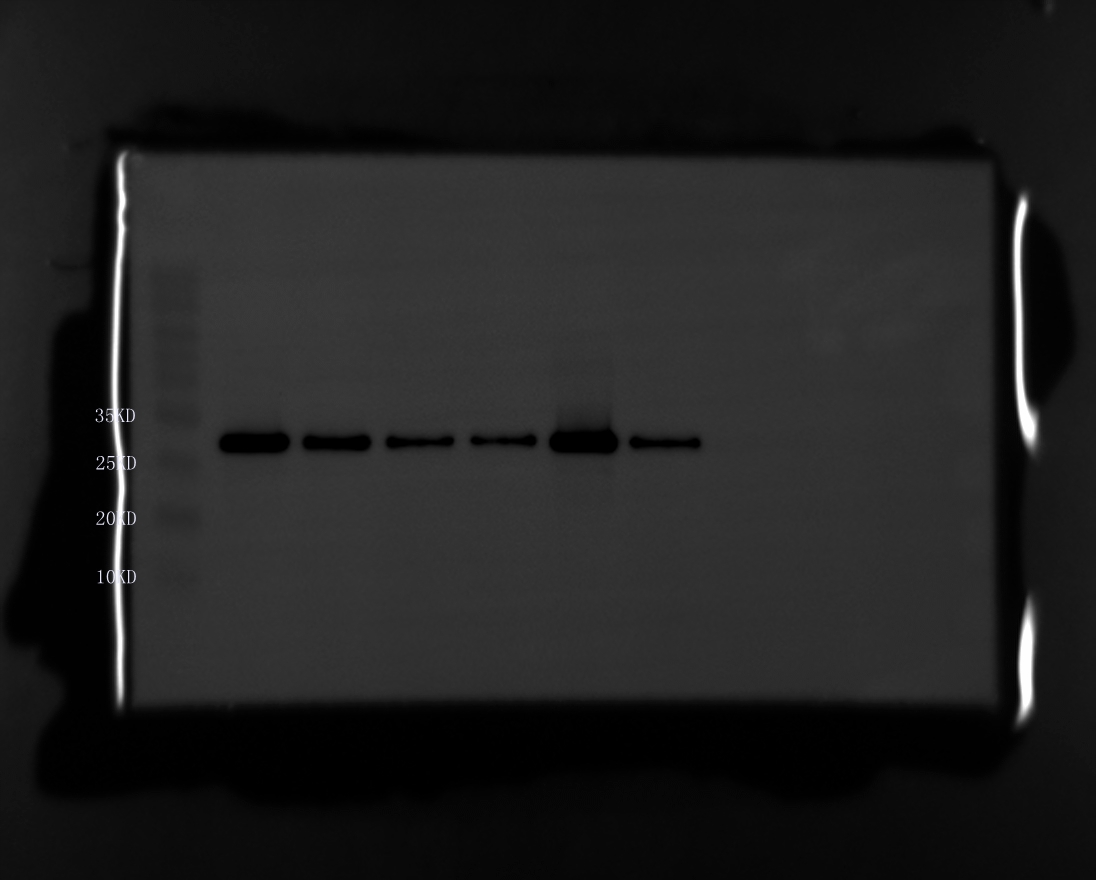

Supplement: S1 File — (ZIP) [file pone.0277719.s001.zip › original blot images/Bcl-2(2).jpg]

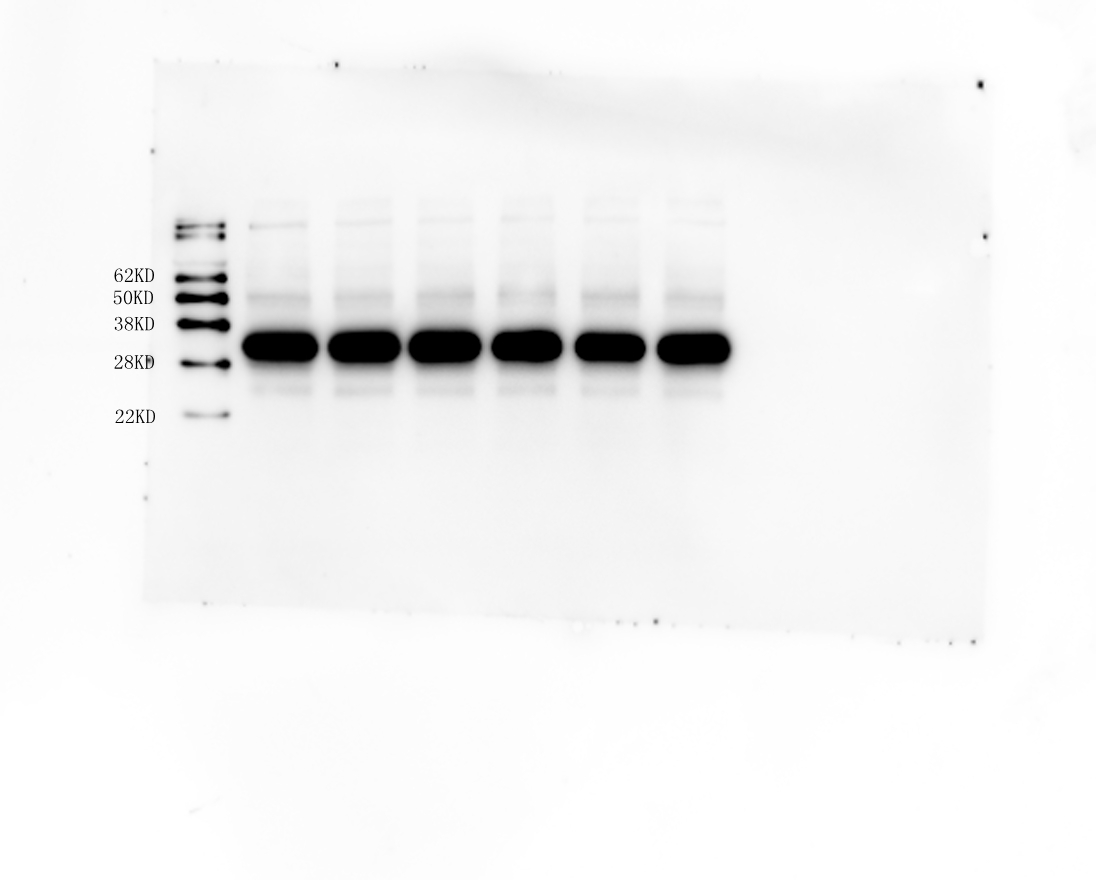

Supplement: S1 File — (ZIP) [file pone.0277719.s001.zip › original blot images/GAPDH.jpg]

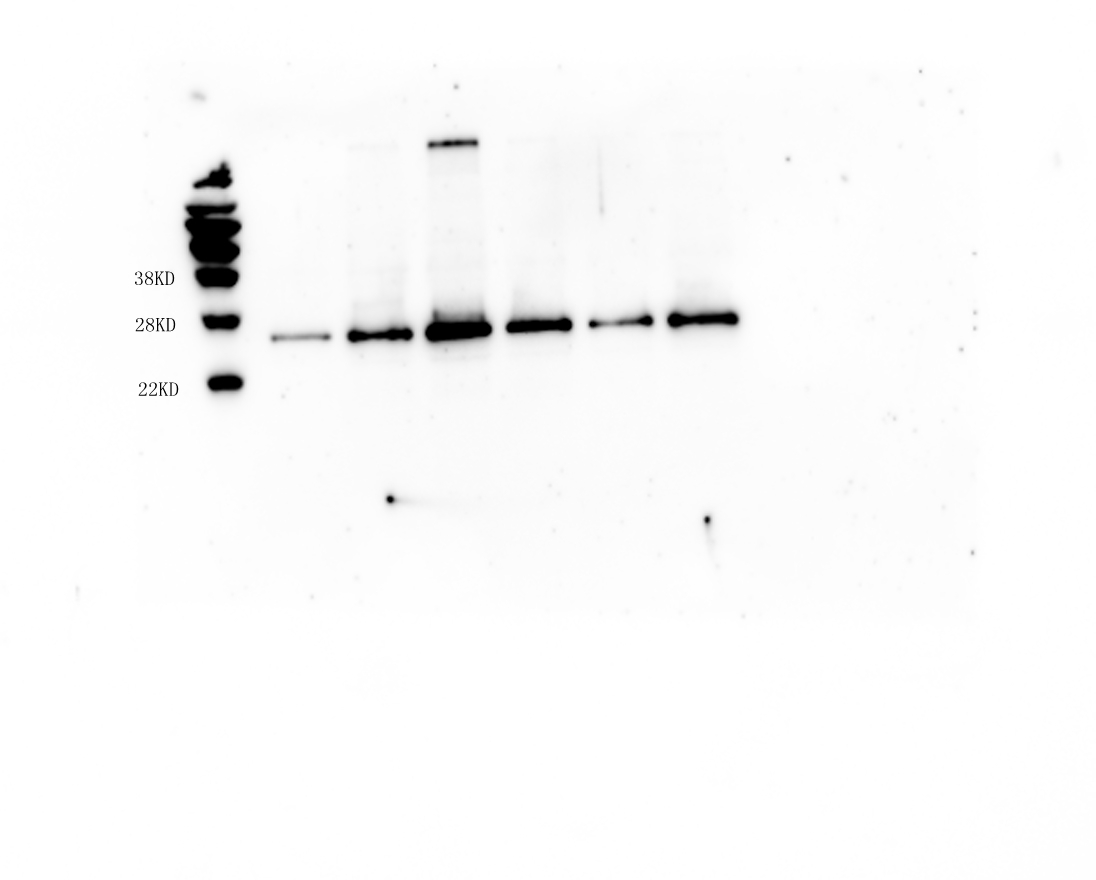

Supplement: S1 File — (ZIP) [file pone.0277719.s001.zip › original blot images/IL6.jpg]

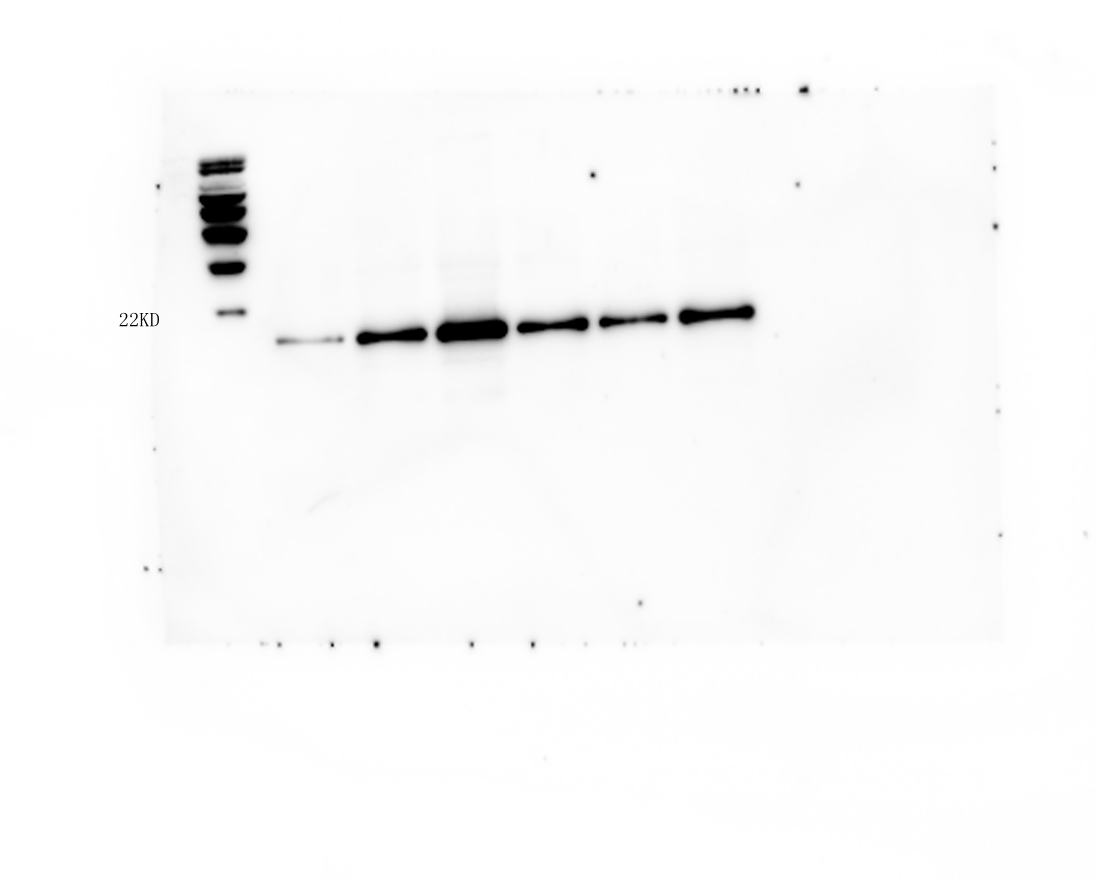

Supplement: S1 File — (ZIP) [file pone.0277719.s001.zip › original blot images/IL8.jpg]

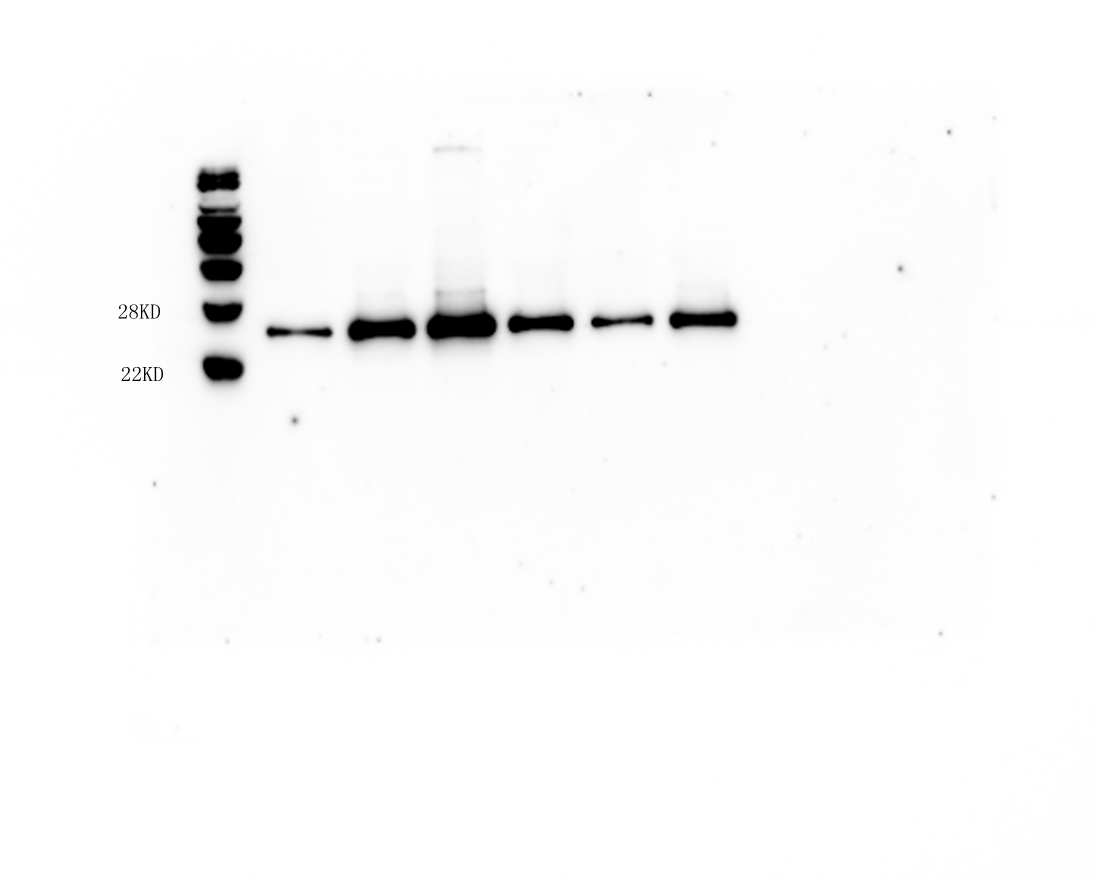

Supplement: S1 File — (ZIP) [file pone.0277719.s001.zip › original blot images/TNF-A.jpg]

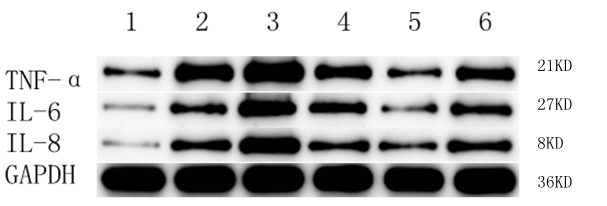

Supplement: S1 File — (ZIP) [file pone.0277719.s001.zip › original blot images/cropped and adjusted image 1.jpg]

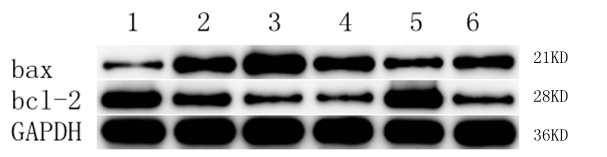

Supplement: S1 File — (ZIP) [file pone.0277719.s001.zip › original blot images/cropped and adjusted image 2.jpg]
