# Supplementary material for: IL-1β knockdown inhibits cigarette smoke extract-induced inflammation and apoptosis in vascular smooth muscle cells
Source: PLoS One. 2023 Feb 15;18(2):e0277719. doi: 10.1371/journal.pone.0277719 (PMC9931126; doi:10.1371/journal.pone.0277719)
Supplement: Attachment — Submitted filename: Response to Reviewers.docx [file pone.0277719.s002.docx]

Response to Reviewers

Dear Editor and Reviewer #1 :

Thank you for carefully reviewing our manuscript previously titled “IL-1β knockdown inhibits cigarette smoke extract-induced inflammation and apoptosis in vascular smooth muscle cells ” for possible publication in the PLOS ONE. We are grateful to reviewer #1 for your effort reviewing our paper and your positive feedback. The summary of our work as written by this reviewer #1 is precise. We have revised the manuscript, highlighting our revisions in red. and have attached point-by-point responses detailing how we have revised the manuscript in response to the reviewers' comments below.

Thank you for your consideration and further review of our manuscript. Please do not hesitate to contact us with any further questions or recommendations.

Yours Sincerely,

Hongfeng Jiang

Department of Geriatrics, Wuhan Fourth Hospital, Affiliated Puai Hospital of Tongji Medical College of Huazhong University of Science and Technology, Wuhan, China

Reviewer #1

**No page numbers. This makes the review complicated.

Response: We marked the page number and line number in the revised manuscript.

Abstract

-page 1, line 20: CSE administion = administration

Response: It was a spelling error and “administion” has been replaced by “administration”.

-page 1, line 23 – The conclusion needs a grammar review (IL-1β involves = IL-1β is involved…and other parts)

Response: The mistakes in grammar has been corrected.

-page 1, KEYWORDS. Words in the title are already keywords.

Response: We added a keyword: atherosclerosis.

Introduction

-Page 2 lines 11 and 12– the word “atherosclerosis” is repeated. Please, be concise.

Response: “atherosclerosis” has been deleted.

- Globally the introduction can be reduced because there are many unneeded words, and some ideas are repetitive. For example, in lines 16 to 25; or lines 26-28… Specifically, the phrase: “IL-1β is essential for vascular inflammation” does not add any new information. The same at page 2, “Many studies support that IL-1β involves in cardiovascular disease”. Details that the authors think are important to understanding their results are better located in the discussion. I recommend revising this part of the manuscript.

Response: This part has been rewritten as: “The cytotoxicity test of the cigarette smoke is commonly conducted using the cigarette smoke condensate and cigarette smoke extract (CSE). Previous study showed the activation of major atherosclerotic key parameters by CSE , such as adhesion molecules intercellular adhesion molecule-1 (ICAM1), vascular cell adhesion molecule-1(VCAM1), selectin E (SEL). Animal studies have suggested that Interleukin-1β (IL-1β) is an important cytokine, whose secretion and activation promotes the formation of atherosclerosis and the instability of vascular plaque [9-11]. The roles of IL-1β in CSE-induced inflammation and apoptosis in vascular smooth muscle cells are rarely known.”

- page 3, line 7: “which is a novel metalloproteinase”. I didn’t understand what “novel” means. It is new in the acknowledgment, right?

Response: Novel means new. PAPP-A is no longer a new metalloproteinase, so “novel” was deleted from the article.

-- page 3, line 20: pro-inflammatory cytolines = cytokines

Response: “Cytolines” was misspelled. It should be cytokines.

-- page 3, lines 24 to 29 and the next page until “We aimed to investigate.. are information more appropriate for the method section.

Response: We have rewritten the paragraph.

Results

-Pg 7, lines 19-20: “To investigate the effect of CSE administration on the expression level of PAPP-A, the concentrations of PAPP-A in culture medium was detected.” Is this phrase needed? Please, be concise.

Response: We agreed with the reviewer and deleted this sentence.

-Page 8, figure 1 legend is incomplete. The authors need to show what CSE, and PAPP-A abbreviations are, so the reader can understand their results without reading the complete manuscript (the same for figures).

Response: Add CSE, and PAPP-A abbreviations According to the reviewer's opinion.

-- Fig 2 shows A and B independent graphics, however, the legend did not clarify which information each figure represents. What is EV? Figures should be explained without text support.

Response: Figure.2A and Figure.2B have been marked. We explained EV in the figure legend.

--page 9: … significantly respectively ... (?); … In contranst,

Response: This sentence lacked a subject and was revised as: In overexpression and knockdown of IL-1β group, the apoptosis rate and Bax protein expression showed significantly respectively increased and decreased compared to model group. In contrast, the expression level of Bcl-2 protein was respectively reduced and increased on overexpression and knockdown of IL-1β group (Fig.3A-D; P < 0.01).

--page 10, figure 3, the phrase “The expression levels of apoptosis-related proteins were detected by Western blot” does not add information and is not appropriate for a legend. I also recommend avoiding the excessive use of the word “significantly”. It is obvious that the mathematics proved that p < 0.05, results are different.

Response: “The expression levels of apoptosis-related proteins were detected by Western blot” was deleted in Fig legend，

--page 10: 2.4 IL-1β “reguglated” ? …. reduced PAPP-A “expresson”(?)

Response: It was a spelling error. reguglated= upregulated, expresson= expression

--page 10: “Then the effect of IL-1β knockdown on PAPP-A mRNA expression was determined.” = Unneeded phrase.

Response: Delete this sentence.

--page 11, fig 4: “The mRNA expression levels were determined by qPCR.” This information is already shown in the method section and does not help to understand the figure results. I also recommend revising the legend, avoiding unneeded words.

Response: “The mRNA expression levels were determined by qPCR.” was deleted.

-- page 11, …significantly “recuded”(?); … might be a “potential”(?) target

Response: IL-1β knockdown significantly downregulated TNF-α, IL-6, and IL-8, which implied that IL-1β cytokine could be a pharmacological target to prevent atherosclerosis induced by cigarette smoke.

--page 12: … inflammation “aossociated” (?) proteins

Response: It should be” Inflammation-related proteins” or “cytokines”.

--page 13: The main “companet”(?) of, …which is “an” critical factor..

Response: It was a spelling error. “The main components of……which is a critical factor….”

--page 13: The authors attributed nicotine to the cause of the increased risk of atherosclerosis. However, they did not show any information from the literature confirming this statement. Moreover, they did not discuss other toxic compounds with more pro-inflammatory effects than nicotine. Most of them are carcinogenic too. Is there any study proving the cardiotoxic effect of nicotine? How could other cigarette compounds be responsible for the pro-inflammatory effects of cigarette smoke? If the authors think that nicotine is the most important source of the cardiotoxic effect why they did not treat the cells with this isolated compound? Reference 20 does not state that “Cigarette smoke is the leading cause of atherosclerosis (20)”.

Response: The reviewer's queries is reasonable. The view that nicotine was attributed to the cause of increased risk of atherosclerosis was not relevant to this study, and the heart toxicity of nicotine was not studied in detail in this study page, so we rewroted this paragraph. Reference 20 has also been replaced.

--page 14: … mitochondrial “proteions”. ... knockdown and “inhibiton”

Response: It was a spelling error. proteions = proteins	inhibiton =inhibition

--page 15: … “expresson” IL-1β and PAPP-A; … and “atherosclerosi.”; … in the “furture.”

Response: It was a spelling error. expresson=expression	atherosclerosis= arteriosclerosis	furture = future

Conclusion:

-- please revise: CSE “could induce” apoptosis …(?). According to their results, the authors can confirm that “CSE induces apoptosis and …”.

Response: CSE induces apoptosis and inflammatory response with increased PAPP-A expression in rat thoracic aortic smooth muscle cells.

-- IL-1β “is involved” in CSE-induced PAPP-A expression and apoptosis.

In this section, the authors repeated information from their results without a more conclusive statement. Indeed, they showed in vitro that cigarette smoke can show a cardiotoxic effect related to inflammatory mechanisms, more specifically to the IL-1β expression. Additionally, I do not agree with the statement that “IL-1β might be considered as a potential clinical target in the treatment of cardiovascular diseases” but a target for preventing cardiovascular diseases caused by cigarette smoking. It is a challenging question to think about.

We agree with the reviewer's suggestion. IL-1β might be considered as a pharmacological target for preventing cardiovascular diseases caused by cigarette smoking.

Response: Thank the reviewer for the accurate summary of our work and brings forward constructive questions. The paragraph was revised as: Our study in vitro that CSE can show an atherosclerotic effect related to inflammatory mechanisms, more specifically to the IL-1β expression. IL-1β might be considered as a pharmacological target for preventing cardiovascular diseases caused by cigarette smoking.
